# Effect of facial and nasolabial asymmetry on perceived facial esthetics in children with non-syndromic cleft lip and palate

**DOI:** 10.1007/s00784-024-05839-4

**Published:** 2024-07-26

**Authors:** Philipp Kauffmann, Johanna Kolle, Anja Quast, Susanne Wolfer, Boris Schminke, Philipp Meyer-Marcotty, Henning Schliephake

**Affiliations:** 1 Department of Oral and Maxillofacial Surgery, University Medical Center Göttingen, Göttingen, Germany; 2 Department of Orthodontics, University Medical Center Göttingen, Robert-Koch-Straße 40, 37099 Goettingen, Germany

**Keywords:** Cleft lip palate, Facial esthetics, Asymmetry, Stereophotometry

## Abstract

**Objective:**

The aim of the present study was to objectively assess the degree of residual facial asymmetry after primary treatment of non-syndromic unilateral cleft lip and palate (UCLP) in children and to correlate it with subjective ratings of facial appearance.

**Materials and methods:**

Stereophotometry was used to record the faces of 89 children with UCLP for comparison of cleft and non-cleft sides up to 5 years after primary cleft closure. Root mean square values were calculated to measure the difference between the shape of cleft and non-cleft sides of the face and were compared to controls without a cleft lip. The Asher-McDade Aesthetic Index (AMAI) was used for subjective rating of the nasolabial area through 12 laypersons.

**Results:**

Children with a cleft lip (CL) showed no significant difference in RMS values compared to controls. Significant differences occurred when the evaluation was limited to the nasolabial area, however only in patients with cleft lip alveolus (CLA) and cleft lip palate (CLAP)(*p* < 0.001). In contrast, subjective ratings showed significantly higher values for all three cleft severity groups (CL, CLA, CLAP) compared to controls (*p* < 0.001). There was a non-linear correlation between the RMS (root mean square) values and the AMAI score.

**Conclusions:**

Even non-significant discrete objective deviations from facial symmetry in children after primary closure of UCLP are vigilantly registered in subjective ratings and implemented in the judgement of facial appearance.

**Clinical relevance:**

3D stereophotometry is a usefull tool in monitoring asymmetry in patients with a cleft.

## Introduction

One of the aims of the treatment of individuals with cleft lip and palate (CLP) is to achieve a harmonic facial appearance through a multidisciplinary approach. An essential characteristic of a harmonic human face is facial symmetry that is affected on all levels of CLP treatment by presurgical measures, timing and technique of surgical cleft closure and postsurgical orthodontic treatment. Although it is a common fact that human faces are not perfectly symmetrical, there is a certain degree of facial asymmetry that is critically noted and negatively judged with respect to facial esthetics [1, 2]. In CLP patients, symmetry of the nasolabial area is particularly difficult to achieve which may result in considerable social distress and the risk of social exclusion and disadvantage [1, 3].

Despite the importance of facial symmetry as clinical outcome parameter in cleft care, the evaluation of facial esthetics in CLP patients based on facial symmetry is neither easy nor well established [4]. On the one hand, objective assessment of facial symmetry requires the evaluation of morphological differences between the operated and the non-operated sides of the face. On the other hand, perception of esthetic dimensions of facial symmetry has to be assessed using validated subjective rating systems that add the esthetic dimension to the results of objective symmetry assessments.

Objective measurement methods for the evaluation of facial symmetry vary greatly using two- or three-dimensional image data for evaluation [5]. The most frequently used imaging modality in recent years has been photogrammetry / stereophotometry creating 3D surface data of the recorded faces, which has already been described as early as 1995 [6]. A frequent problem of the evaluation of the recorded 3D images is the amount and complexity of the derived data. Many approaches have tried to reduce this complexity by manually defining points, distances and angles as well as symmetry planes for comparison of cleft vs. non-cleft sides in 2D and 3D pictures [7–10]. Manual identification of landmark points may, however, give rise to a certain degree of operator bias. Hence, when 3D pictures were used, it has been advocated to make full use of the 3D data sets by a 3D shape analysis with an automated mapping strategy that minimizes operator bias [5].

Subjective ratings of facial symmetry and esthetics in CLP patients also offer a wide range of indices that provide different degrees of validation and focus on different features based on 2D photographs [4]. Among these scoring systems, the Asher-McDade Aesthetic Index (AMAI) has proven to be a reliable and reproducible method to reflect the subjective perception of the nasolabial appearance and esthetics [11, 12]. However, it should be kept in mind that when 3D images are used instead of standard photographs, raters need to be trained appropriately to be able to evaluate the additional information provided by the 3D images [13].

It was thus the aim of the present study to objectively assess the nasolabial symmetry in children with unilateral cleft lip and palate (UCLP) with different degrees of CLP severity after primary cleft closure using 3D stereophotometry with automated mapping and to correlate the results with subjective ratings of nasolabial appearance in these patients.

## Material & methods

Nasolabial asymmetry was assessed in a cross-sectional evaluation on 89 children (male = 55 / female = 34) chosen out of 493 individuals listed in the data bank of the Craniofacial & Cleft Care Center of the University Medicine of Goettingen. The following selection / inclusion criteria were applied: (i) non-syndromic unilateral CLP; (ii) no secondary surgery (e.g. lip revision); (iii) no adjunctive treatment (e.g. orthodontic therapy); (iv) stereophotometric 3D data set of the patient correctly positioned showing complete facial soft tissues at rest. Using these criteria, 25 cases(male = 18 / female = 7) with unilateral cleft lip (CL), 21 cases (male = 8 / female = 13) with cleft lip / alveolus (CLA) and 43 cases (male = 29 / female = 14) with cleft lip / alveolus / palate (CLAP) were included. The mean age of the included patients was 6.5 ± 1.1 years.

All patients had been treated according to the same presurgical / surgical protocol: (i) presurgical therapy using NAM appliances starting at 1 month of age; (ii) cleft lip closure with primary rhinoplasty and periosteoplasty for closure of alveolar clefts (if applicable) at the age of 4–6 months; (iii) cleft palate closure in two steps at the age of 12–15 months. 11 children (male = 7 / female = 4) with isolated cleft palate without any involvement of lip/nose/alveolus were used as controls.

For esthetic rating of the nasolabial region, 36 patients (12 patients each with cleft lip, cleft lip / alveolus, cleft lip / alveolus / palate) were randomly selected from the 89 patients whose pictures had been used for objective assessment of nasolabial asymmetry and evaluated together with the controls. The sample size of patients undergoing aesthetic evaluation was planned according to the sample size used in previous studies [19, 22]. It compares also quite well to the sample size of a recently published on the esthetic rating of the nasolabial area using 3D [14].

The study protocol had been approved by the local ethical committee (Registration Number 21/4/20) and the study had been performed according to the principles of the Declaration of Helsinki (https://www.wma.net/policies-post/wma-declaration-of-helsinki-ethical-principles-for-medical-research-involving-human-subjects/).

### Assessment of asymmetry

**Fig. 1:**
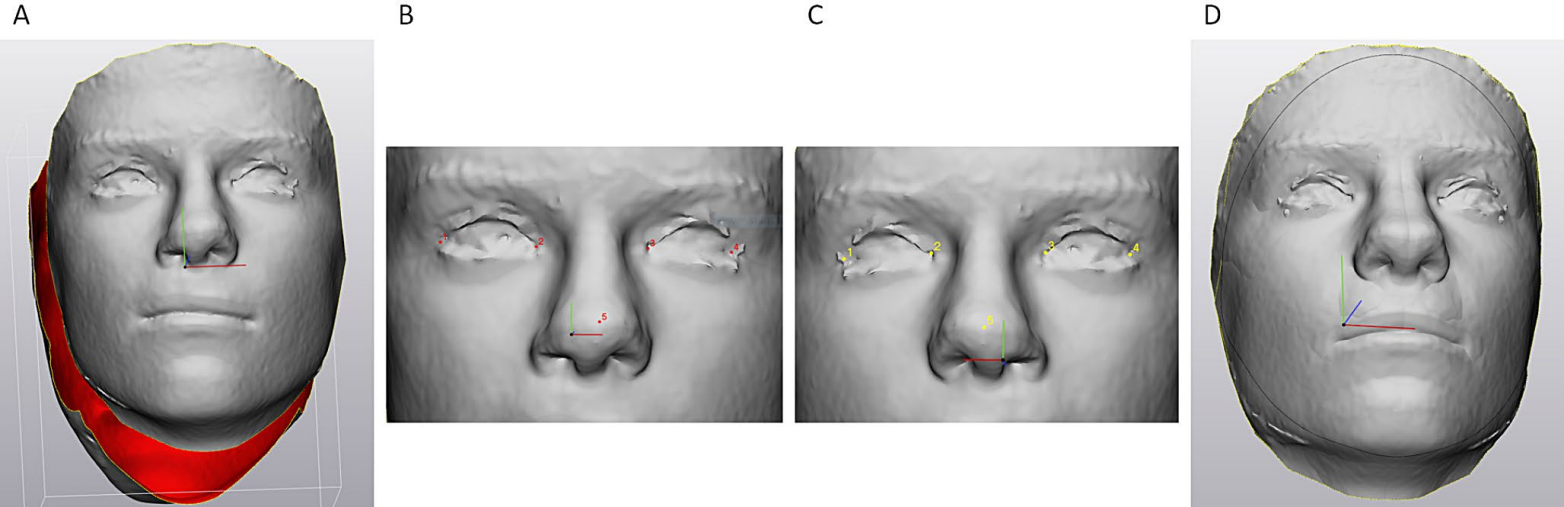
**A)** Original 3D surface image **B&C)** Gross construction of a symmetry plane using medial and lateral canthus and the nose tip for mirroring **D)** Final overlay of mirrored images after defining a markerfree symmetry plane using ICP. (informed consent of patient existing)

**Fig. 2:**
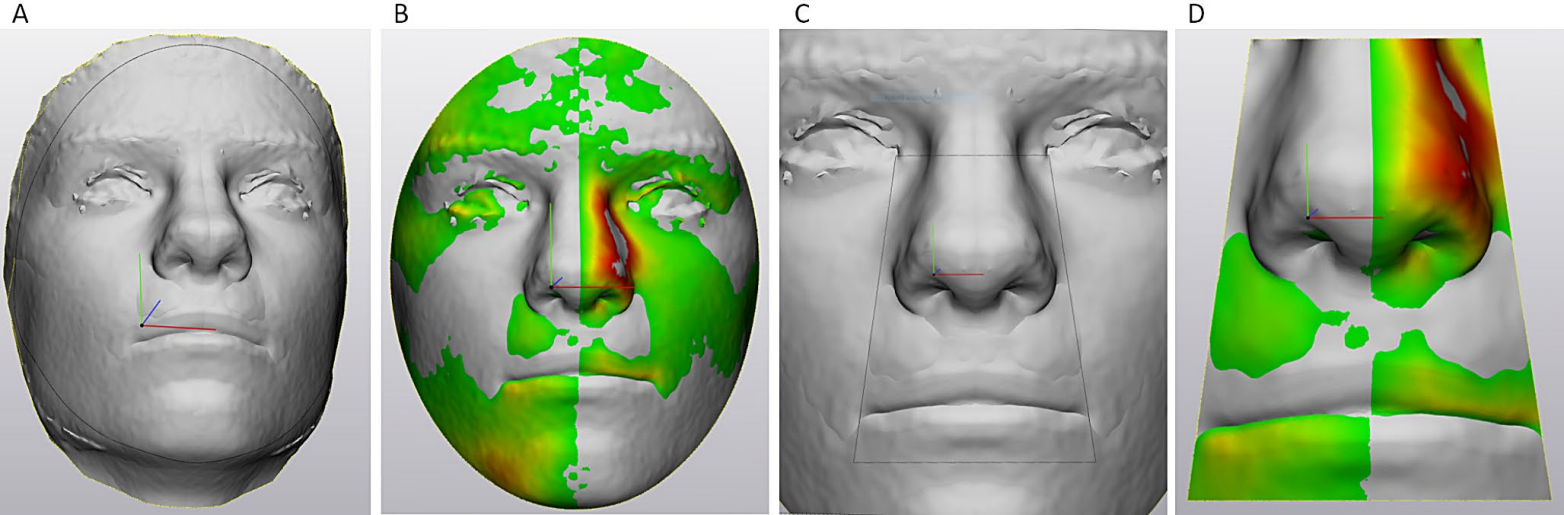
**A&B)** Part comparison analysis (PCA) of the whole face **C&D)** Part comparison analysis (PCA) of the nasolabial trapezoid. (informed consent of patient existing)

Three-dimensional images of the patients with their facial soft tissues at rest were recorded using a stereo-camera system (Vectra-M5-360 camera, Parsippany, NJ, USA). The recorded data were reconstructed into 3D images using Vectra software (Vers 5.7.2, Canfield Scientific) for triangulation of surface points. Reliability and accuracy of the Vectra System had been previously reported [15]. Mean differences between linear measurements and 3D measurements in the facial region ranged between − 0.06 and − 0.37 mm), indicating slightly but insignificantly larger dimensions of the 3D data. For evaluation of facial asymmetry, a symmetry plane was constructed by approximation of the original image and its mirrored version through an iterated-closest-point algorithm (ICP-algorithm, Besl & McKay 1992) using 3-matic software (Vers. 16.0, Materialise, Leuven, Belgium). This resulted in the marker-free definition of a median sagittal plane that could be used for evaluation of morphological differences between the operated and the non-operated sides. Differences between the two sides were assessed by mirroring the 3D-data of the non-operated side at the symmetry plane with subsequent overlay of the newly created “ideal symmetry” image with the original image using n-point registration for gross orientation (Fig. 1A through C) and the ICP algorithm for final optimized overlay (Fig. 1D). Subsequent part comparison analysis (PCA) resulted in display of mean distances between the surface triangles of both data sets in a color coded map (Fig. 2A and B); additionally the root mean square distance (RMS) values between the surface triangles of the mirrored “ideal” and the original image were calculated in mm as a numeric measure for facial asymmetry. Both PCA and RMS were performed on data sets of the whole faces and the nasolabial areas that had been cropped according to Desmedt et al. (2015) using two horizontal and two oblique lines defining a trapezoid that contained the nasolabial area (Fig. 2C and D).

### Assessment of facial esthetics

**Fig. 3:**
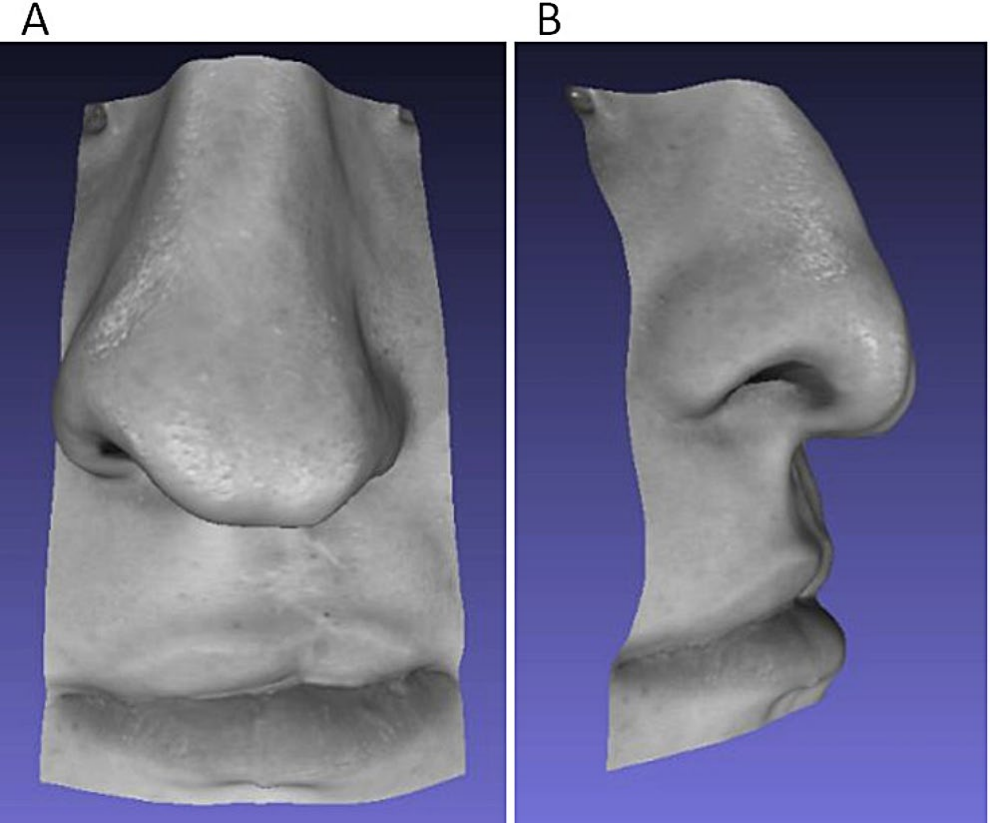
Nasolabial trapezoid of a patient with a CLP exhibiting strong asymmetry. Images could be manipulated in all direction showing e.g. frontal **(A)** and lateral **(B)** views. (informed consent of patient existing)

Facial esthetics were evaluated using the Asher-Mcdade Aesthetic Index (AMAI) that assessed (i) nasal form (frontal view), (ii) deviation of the nose (frontal view), (iii) shape of the vermilion border and (iv) the nasal profile including upper lip (lateral view) using a 5-point scale for each feature [16]. The assessment was performed by 12 adult lay volunteers (male = 6, female = 6, age: 33.4 y ± 14 years), who had been blinded to the diagnosis (CL, CLA, CLAP, control) and had been calibrated using five data sets for exercise. The pictures used for aesthetic assessment displayed the nasolabial trapezoid identical to those used for assessment of asymmetry. The textured image data of this area had been saved as *object* (OBJ) data files and displayed as grey images using Zoner Photo Studio^®^ (Version 12, Zoner Inc., https://www.zoner.com) (Fig. 3). Images were assessed in random order, all images could be manipulated in all directions on the screen during evaluation using MeshLab (Version 2020.12, Consiglio Nazionale delle Ricerche (Rome, Italy)).

### Statistical evaluation

Root Mean Square (RMS) values were calculated for the whole face and the nasolabial trapezoid of each patient and tested for normal distribution using Shapiro-Wilk-tests (IBM SPSS Statistics^®^ (Version 28.0.0.0) IBM Corp. (Armonk, NY, USA)). These tests indicated a significant deviation from normal distribution (*p* < 0.05). Hence, mean values calculated for cleft severity groups (CL, CLA CLAP) and controls and were compared using Kruskal Wallis- tests at a level of significance of *p* < 0.05.

Asher-Mcdade Aesthetic Index (AMAI) values were tested for normal distribution in an identical manner as described above showing normal distribution (*p* > 0.05). AMAI mean values calculated for the cleft severity groups and controls were thus compared using ANOVA tests at a level of significance of *p* < 0.05.

An association between subjective rating and objective asymmetry was assessed by calculating a Spearman-Rank correlation between RMS values and AMAI values with *p* < 0.05 being considered as significant. In case the data indicated a non-linear connectedness, curve estimation regression models were applied.

## Results

**Fig. 4:**
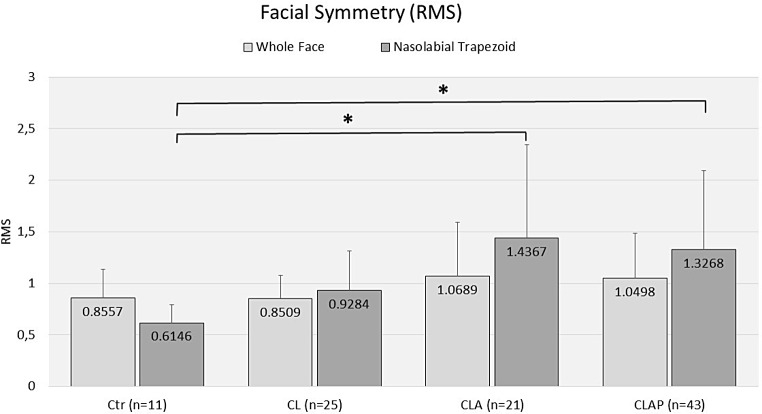
RMS values for comparison of asymmetry of the whole face and the nasolabial trapezoid

The calculation of RMS values for the whole face showed almost equal mean values for the controls and the CL patients (0.8557 ± 0.2798 and 0.8509 ± 0,2269, respectively), whereas the mean values of the CLA and CLAP groups were higher (1.0689 ± 0.5201 and 1.0498 ± 0.4341, respectively). However, differences between the four groups for the whole face were not significant (*p* = 0.267) (Fig. 4). The RMS values calculated for the nasolabial trapezoid exhibited the lowest mean value for the control groups (0.6146 ± 0.1759), followed by the mean value of the CL patients (0.9284 ± 0.3861). Mean values of the CLA group (1.4367 ± 0.9056) and the CLAP group (1.3268 ± 0.7659) were significantly higher than the mean value of the control group (*p* < 0.001). Differences between the mean values of controls and the CL group (*p* = 0.089) as well as the CL group and the CLA and CLAP groups were not significant (*p* = 0.096 and 0.110, respectively) (Fig. 4).

**Fig. 5:**
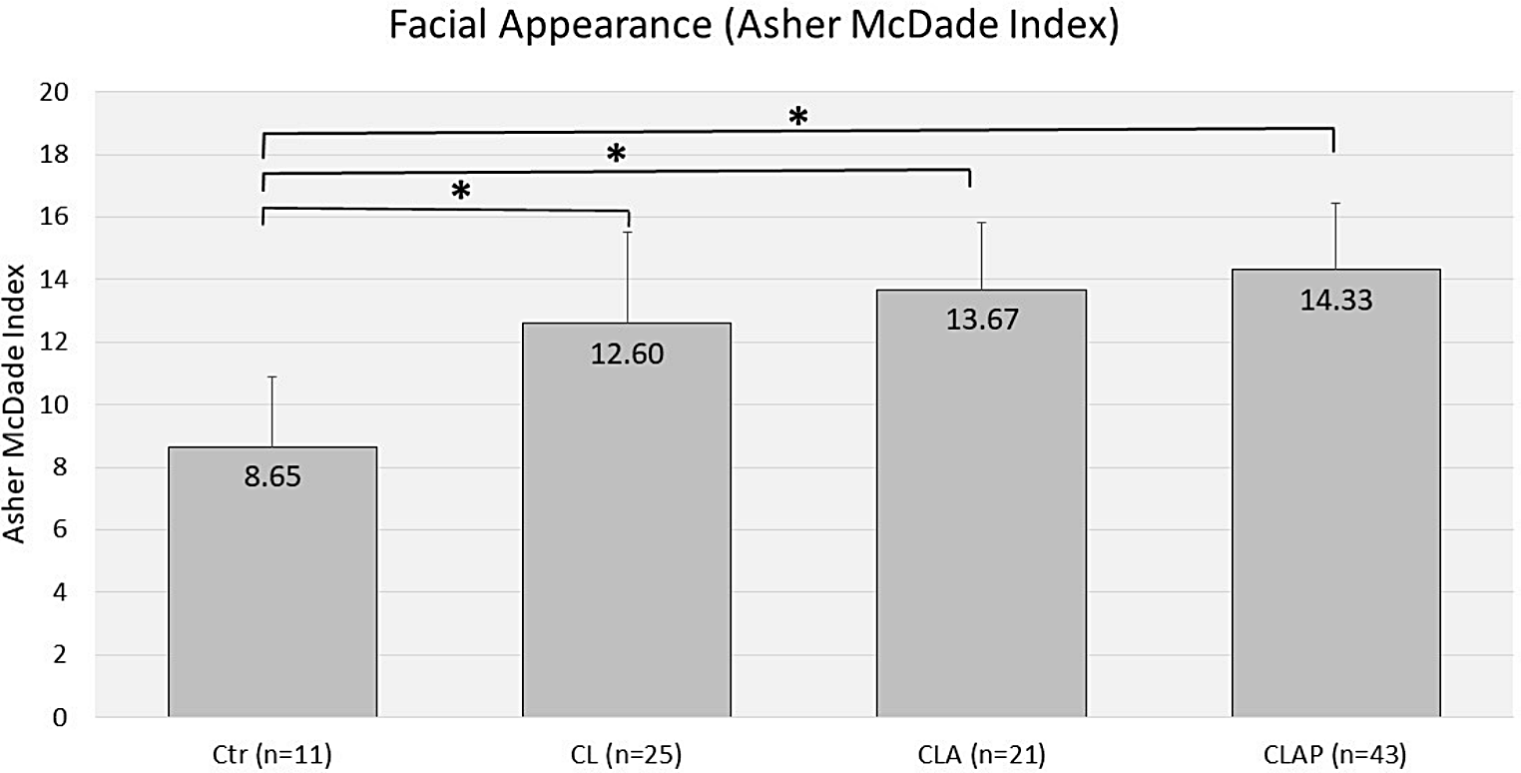
Asher Mcdade Aesthetic Index (AMAI) of cleft groups and controls

Assessment of nasolabial esthetics using the AMAI showed the lowest mean value for the controls (8.65 ± 2.25), whereas the mean values of all cleft groups were significantly higher (CL: 12.60 ± 2.93; CLA 13.67 ± 2.14; CLAP: 14.33 ± 2.10) with p-values < 0.001 for all three groups (Fig. 5).

**Fig. 6:**
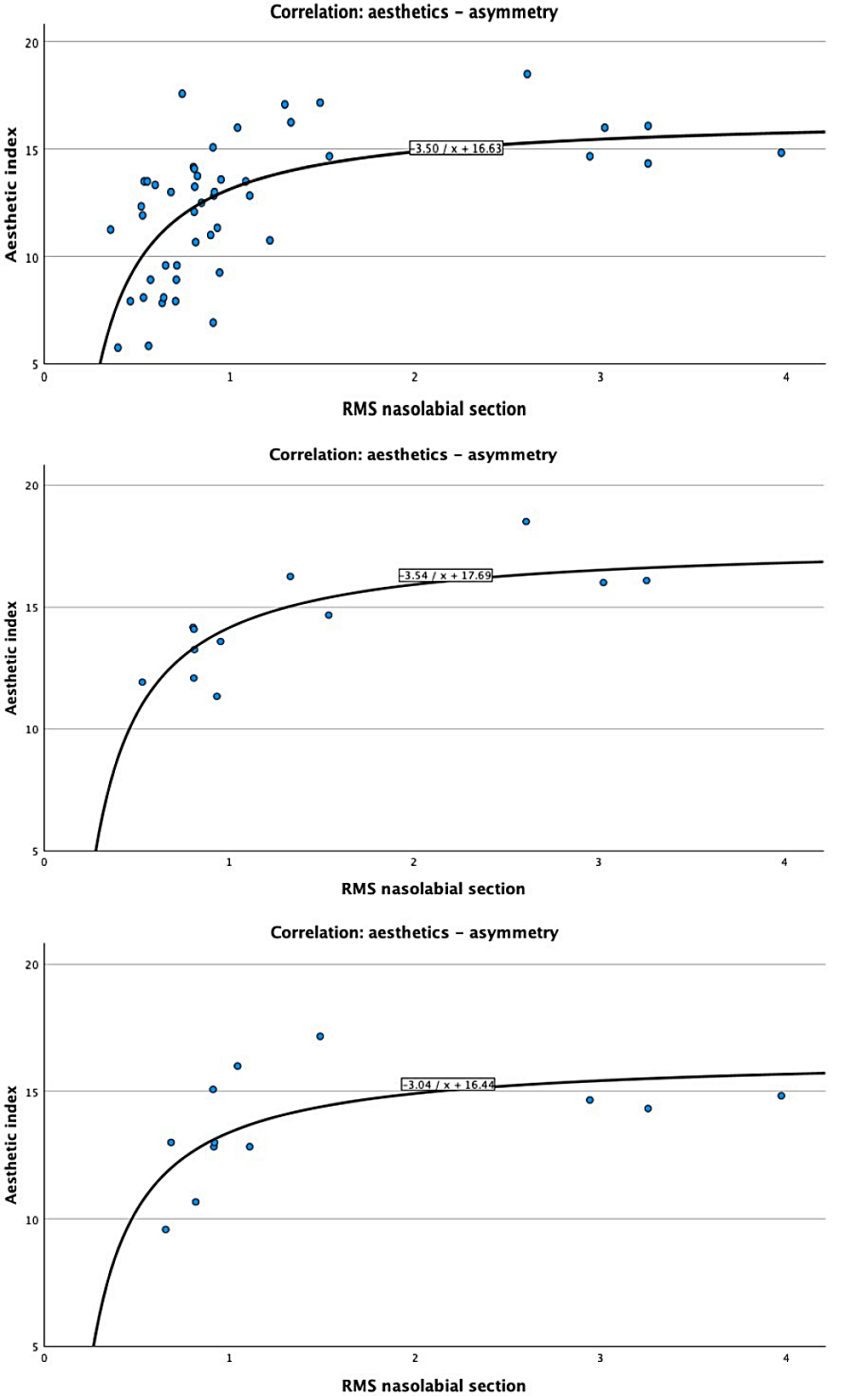
**(A)** Correlation between aesthetics and asymmetry. Shows all 48 average values of the aesthetic index depending on the associated degrees of asymmetry. The graph shows the inverse model equation of curve fitting with the equation y=-3.499/x + 16.630. R² = 0,378 **(B)** Correlation between aesthetics and asymmetry. Shows 12 average values of the aesthetic index depending on the associated degrees of asymmetry. The graph shows the inverse model equation of curve fitting with the equation y=-3,54/x + 17,69.R² = 0,637 C) Correlation between aesthetics and asymmetry. Shows 12 average values of the aesthetic index depending on the associated degrees of asymmetry. The graph shows the inverse model equation of curve fitting with the equation y= -3,04/x + 16,44. R² = 0,379

Calculation of the Spearman Rank correlation showed a significant correlation between the RMS values and the AMAI values with a correlation coefficient ρ = 0.635 (*p* < 0.001), when all values were included into the analysis (Fig. 6A). As plotted data of all combined groups indicated the need for a non-linear curve estimation model, an inverse curve estimation regression model was applied with y = -3,499 / x + 16,630. (R² = 0.378, *p* < 0.001). For the individual cleft severity groups, inverse curve estimation also identified significant modelling parameters for the CLA (y = -3.042 / x + 16.437. (R² = 0.379, *p* < 0.033) and CLAP group (y = -3.545 / x + 17.686. (R² = 0.637, *p* < 0.002) (Fig. 6B and C).

## Discussion

The present study has assessed facial asymmetry in UCLP patients with different CLP severity using a landmark-independent method for stereophotometric comparison of cleft and non-cleft sides of patients’ faces. The approach of automated mapping without the manual definition of landmarks has been considered to be preferable when 3D surface data of facial soft tissues are assessed in order to minimize operator induced bias during the evaluation [5]. Moreover, surface based registrations have shown to be highly reproducible at a higher degree of precision [17–19]. Nevertheless, it should be kept in mind that asymmetric distortion of facial soft tissues in cleft patients also may affect the non-cleft side. In particular, the nasal tip and the nasal septum are frequently deviated to the non-cleft side. Even the most precisely constructed symmetry plane thus will mirror this deviation and thereby may introduce some bias into the assessment of asymmetry by providing a “contaminated” reference shape for the operated side that is not completely unaffected by the deformity.

The results of the present study have shown that there is an increasing degree of measurable deviation of surface contours between the operated and the non-operated side with increasing severity of cleft manifestation when the nasolabial area is considered. As it may have been expected, this difference in shape has become significant when facial bones are involved in CLA and CLAP patients indicating the effect of supporting skeletal structures on the shape of the overlying facial soft tissues already at this early age. Admittedly, the age range of the evaluated cohort does not allow for a conclusive discussion of the effect of the development of skeletal anatomy on the shape of facial soft tissues. However, this age group has been chosen deliberately to assess the effect of primary measures for cleft closure as purely as possible. Patients at higher age commonly undergo secondary surgery as well as orthodontic therapy that may obscure the effect of presurgical therapy and the primary surgical intervention for cleft closure.

The present results in part parallel those of Desmedt et al. [20], who had used very similar means of evaluation in a comparably composed cohort of CLP patients, but at a 5 years higher age on average. In contrast to the present results, their data indicated a significant difference in asymmetry also between CL-only patients and the CLA- and CLAP-groups suggesting that the degree of asymmetry may increase during further growth in childhood giving rise to the necessity for revisional surgery in the nasolabial trapezoid at adolescence.

The difference between the shape of the cleft and the non-cleft side became non-significant when the whole face was included into the evaluation, which may be explained by a “diluting” effect of the peripheral facial regions that exhibit lesser degrees of asymmetry. Comparison of changes in the shape of the facial soft tissues 3 months before and 8 months after primary closure of cleft lips has shown that only the nasolabial area and the cheeks have shown highly significant changes whereas the forehead, eyes, lower lip and chin did not differ significantly [21]. This effect is stronger in the present study than it has been observed in previous studies [22, 23]. The contradictory results in the present study may be accounted for by the fact that we have used automatic mapping of hundreds of surface points that have a stronger “diluting” effect in the periphery of the face than the 28 anatomical landmarks defined in the study of Hood et al. (2003) [22]. The difference to the results of the study of Meyer-Marcotty et al. (2010b) may be explained by the fact that the authors had evaluated adolescents and adults, where cleft associated differences in skeletal development and facial growth could have resulted in significant asymmetry of the whole face and not only the nasolabial area [23]. Interestingly, evaluations of younger age groups of cleft patients employing comparable methodology have produced results that parallel those of the present study [14].

Assessment of facial appearance using the Asher-Mcdade Aesthetic Index (AMAI) in the present study had shown a significant difference between all faces affected by a cleft lip and those without. Increasing severity of cleft manifestation has been associated with a gradual but non-significant increase in AMAI scores. Interestingly, the difference between the controls and the cleft-lip-only (CL) group was much clearer when the faces had been subjectively rated using the AMAI than in the objective assessment of asymmetry, where no significant difference in mean RMS values between controls and the CL- group had been found. This may be explained by the basic problem of defining a shape through a numeric parameter. Low average RMS values as an objective measure of asymmetry may indicate a very similar shape but may as well obscure major albeit focused surface deviations associated with a punctually distorted shape in combination with otherwise very low RMS values. This punctually distorted shape will be of course noted and judged in a subjective rating system for facial perception. This is probably particularly true in the nasolabial area, as this is the area where unconscious assessment of visual characteristics of one another starts in the beginning of human interactions [24] and even small asymmetric deviations from “average appearance” will most likely be noted rapidly and perceived as decrease in attractiveness [25]. The present results suggest that this also true when faces of children with minor objective cleft related deformity are looked at.

This vigilant registration and negative judgement of minor objective deviations from symmetry is reflected by the non-linear relation between the objective RMS values and the AMAI scores, where particularly the lower RMS values were associated already with high AMAI scores whereas high RMS values have not led to a substantial increase in the AMAI scores. To this end, the results are indirectly supported by those of Desmedt et al. (2015) [20], who had used linear regression analysis without curve estimation modelling and found no significant correlation in more severe forms of CLP [20].

While subjective rating scores reflect the conscious element of facial perception, an important measure to evaluate the initial and mostly unconscious perceptions of facial characteristics has been the analysis of the gaze pattern through eye tracking studies [2, 26, 27]. Analysis of gaze data indicated that the eyes typically scan the triangle between the eyes, the nose and the mouth when faces are looked at with initial fixation on the eyes and subsequent focus on the nose and mouth. This gaze pattern is altered when faces with secondary cleft lip deformity are looked at shifting intervals of fixation away from the eyes towards nose and mouth [26–28]. The deviation in gaze pattern may be subject to changes, depending on age [29] and whether the observer is affected by the congenital deformity her / himself [2]. Despite these observer induced alterations in eye movement patterns, a correlation between the severity of cleft deformity and changes in gaze duration pattern and scan path has been found and a threshold value has been hypothesized below which cleft related deformities may not be realized any more [24]. Looking at the non-linear correlation between objective asymmetry data and subjective ratings, more research will be needed into the unconscious perception of secondary cleft deformity and the objective degree of asymmetry to establish such a relationship.

## Conclusions

The present study has shown that pre-surgical and surgical treatment of unilateral cleft lip and palate (UCLP) has been associated with a moderate degree of asymmetry in children with a significant difference between cleft- and non-cleft side in more severe manifestations of UCLP. Despite a non-significant difference in objective asymmetry in cleft lip only patients, the subjective esthetic ratings had resulted in significantly worse scores indicating that also small asymmetric variations in shape in the nasolabial area of children are vigilantly registered and implemented in the judgement of facial appearance.
